# Development of an evaluation framework for robotic total mesorectal excision videos: a review and comparison of medical professional and public video resources

**DOI:** 10.1007/s00384-025-04914-w

**Published:** 2025-05-24

**Authors:** Zohaib Arain, Michael G. Fadel, Aksaan Arif, Henry Douglas Robb, Bibek Das, Liam Poynter, Christos Kontovounisios, Hutan Ashrafian, Daniel Lawes, Matyas Fehervari

**Affiliations:** 1 Department of General Surgery, Frimley NHS Foundation Trust, Camberley, UK; 2 Department of Surgery and Cancer, Hammersmith Hospital Campus, Imperial College London, Du Cane Road, London, W12 0NN UK; 3 School of Medicine, Imperial College London, London, UK; 4 Department of Colorectal Surgery, Maidstone and Tunbridge Wells NHS Trust, Kent, UK; 5 2nd Surgical Department, Evaggelismos Athens General Hospital, Athens, Greece; 6 Department of Colorectal Surgery, Chelsea and Westminster Hospital NHS Foundation Trust, London, UK; 7 Department of Colorectal Surgery, Royal Marsden NHS Foundation Trust, London, UK; 8 Department of Bariatric Surgery, Maidstone and Tunbridge Wells NHS Trust, Kent, UK

**Keywords:** Robotic total mesorectal excision, Educational video, Surgical video platforms, Video quality assessment

## Abstract

**Purpose:**

This study aims to assess the quality of educational surgical videos for robotic total mesorectal excision (TME), across widely used open-source platforms, using a newly designed quality assessment checklist.

**Methods:**

The checklist was developed by using existing society guidelines, such as the European Academy of Robotic Colorectal Surgery, comprising four key sections: (i) usability of the platform, (ii) video component, (iii) intraoperative techniques and (iv) other information (including case presentation and outcomes). Videos were identified using the search terms ‘Robotic TME’ from surgical education platforms (WebSurg, C-SATS and Touch Surgery) and YouTube, between January 2016 and July 2024. All videos displaying robotic TME were reviewed and scored using the quality assessment tool (/12), and the videos across the platforms were subsequently compared.

**Results:**

A total of 113 videos were scored using the checklist: 63 surgical education platform (10 WebSurg and 53 C-SATS) and 50 YouTube videos. The total median checklist score achieved by WebSurg (9 [IQR 8–9] and YouTube videos (8 [IQR 7–10]) was significantly higher than CSAT-S videos (4 [IQR 4–5]; *p* < 0.001). The usability of platform scores for YouTube was significantly higher than WebSurg and C-SATS videos (*p* < 0.001). Scores for video components, intraoperative techniques and other information were higher across WebSurg and YouTube videos when compared to C-SATS (*p* < 0.001); however, there was no significant difference between WebSurg and YouTube for each domain.

**Conclusion:**

The overall educational quality of online robotic TME videos was found to be generally heterogeneous, with WebSurg and YouTube videos demonstrating higher scores based on the checklist. A new quality assessment tool has been proposed for robotic TME videos, which has the potential to improve the reliability and value of published video research.

**Supplementary Information:**

The online version contains supplementary material available at 10.1007/s00384-025-04914-w.

## Introduction

In the last two decades, there has been a rapid growth in robotic surgery [1]. Robotic surgery has several technical advantages over conventional techniques for certain procedures, including three-dimensional visualisation, articulating instruments and enhanced access to complex or narrow anatomical areas, which may potentially improve surgical quality and outcomes [1–6]. It requires a distinct set of skills, and the steps of the surgical procedures can be technically demanding and difficult to learn [7]. Surgical videos are a useful and widely utilised resource for surgical trainees in learning procedures, with those hosted on open-source web pages (e.g. YouTube©) being the most popular [8–10]. For example, Pape-Koehler et al. [11] performed a randomised controlled trial that demonstrated multimedia-based training can significantly improve surgical performance and the understanding of complex temporal and spatial events. This is particularly useful in minimally invasive surgery, which lends itself to the production of audiovisual educational materials.

Surgical videos can be presented in various methods, including academic journals, surgical education platforms (e.g. WebSurg, C-SATS, Touch Surgery) and commercial platforms (e.g. YouTube). WebSurg is a free online learning platform developed by the Research Institute against Cancer of the Digestive System (IRCAD); C-SATS is an online surgical improvement and proficiency platform, part of Johnson & Johnson; and Touch Surgery is a health technology application created by Digital Surgery. However, surgical education and commercial platforms may present videos in variable forms which may introduce heterogenous reporting of videos, particularly due to the lack of a thorough peer review process [12, 13]. This can ultimately reduce the quality, reliability and educational value of the intended learning outcome [14, 15].

In 2019, the LAParoscopic surgery Video Educational GuidelineS (LAP-VEGaS) consensus was published, which set out a checklist guideline for the standardisation of high-quality educational videos for laparoscopic surgery [14]. Since then, numerous studies have used the LAP-VEGaS framework, or a bespoke checklist adapted from it, to evaluate the landscape of available online videos for specific surgical procedures [15, 16]. To our knowledge, no such study has been done to assess the quality of robotic colorectal educational videos. Between 2013 and 2018, the prevalence of robotic colorectal surgery increased fourfold [17], with a more rapid adoption for rectal cancer treatment [18]. Due to the complex anatomical planes in the pelvis, robotic total mesorectal excision (TME) has been increasingly utilised [19].

In this study, we aimed to assess the quality of educational surgical videos for robotic TME on the following open-source platforms: WebSurg, C-SATS, Touch Surgery and YouTube, using an established checklist for the procedure with adapted elements from the LAP-VEGaS consensus.

## Methods

### Search strategy

Four widely used surgical educational video platforms (WebSurg, C-SATS, YouTube and Touch Surgery) were reviewed in this study. Videos were identified using the following search terms: ‘Robotic TME’ or ‘Robotic Total Mesorectal excision’. Inclusion and exclusion criteria were specified for videos from all four platforms and are described as follows:

#### Inclusion criteria

(i) Videos showing part of, or all of, the robotic TME procedure
(ii) Videos uploaded between January 2016 and July 2024 (for YouTube)

#### Exclusion criteria

(i) Robotic TME was not the primary surgical procedure recorded in the video
(ii) Duplicate videos

### Development of video assessment checklist

The European Academy of Robotic Colorectal Surgery (EARCS) published a consensus on standardisation of the robotic TME procedure [20]. This framework established technical standards for operative set-up and procedural steps in order to improve consistency and predictability of clinical outcomes and training [20]. The main steps identified in this framework were as follows: theatre set-up and patient positioning, port positioning, docking, colonic mobilisation (splenic flexure mobilisation and vascular pedicle dissection), pelvic dissection (posterior, upper right lateral and anterior, left lateral planes, specimen extraction and anastomosis, extracorporeal specimen resection and introduction of the circular staple anvil). These steps were summarised and combined to form the following fields in the checklist used for this study:

(i) Port site and patient position demonstrated (either diagrammatically or physically)
(ii) Robotic procedure demonstrated clearly: colonic mobilisation, clipping of the inferior mesenteric artery (IMA) and the inferior mesenteric vein (IMV) and TME
(iii) Anastomosis and leak test both demonstrated

An additional aspect to the checklist used in this study was the evaluation of the educational quality of robotic TME videos. For these elements, the LAP-VEGaS practice guidelines were applied [14]. The following elements of the LAP-VEGaS video assessment tool were included or adapted in the checklist used in this study:

(i) Image quality is appropriate with a constant clear view of the operating field
(ii) Presentation of case: including age, sex, American Society of Anesthesiologists (ASA) physical status classification, indication for surgery, co-morbidities and previous imaging
(iii) Complications/30-day outcome
(iv) Narration/subtitles

One element of the checklist related to the educational quality of the videos, but not specified in the LAP-VEGaS criteria, was formulated by the authors of this study, including educational components such as interactions, questions and visual prompts.

To directly compare the digital platforms and websites hosting the appraised videos in this study, the authors also included the following quality standards:

(i) Accessibility: no login or registration is required to view the video
(ii) Easy to find video (video identifiable using key search terms)
(iii) Easy to locate specific parts of the procedure (presence of timestamps/sections/chapters)
(iv) Video maintaining patient and surgeon confidentiality

**Fig. 1:**
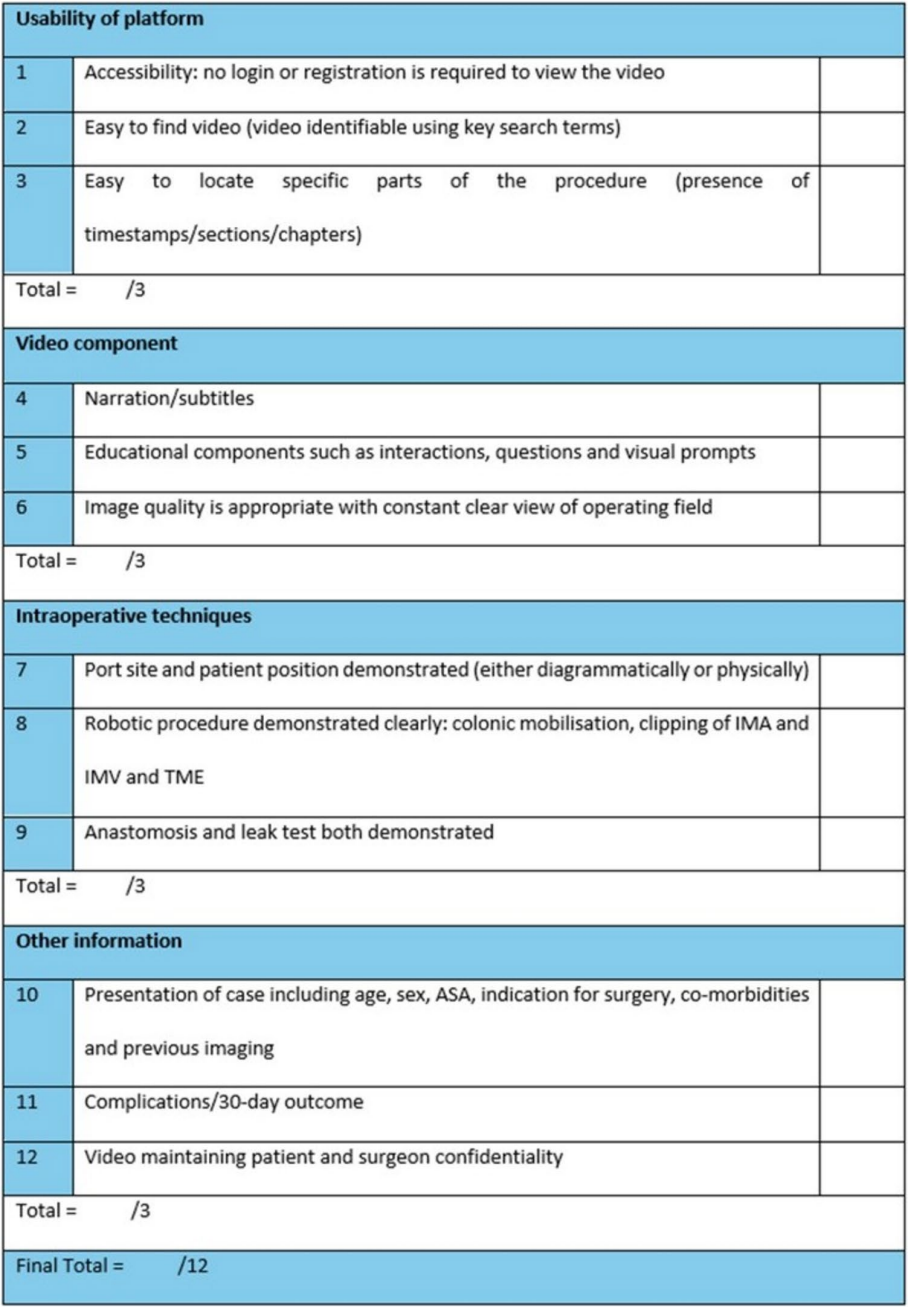
Quality assessment checklist for robotic total mesorectal excision videos. ASA, American Society of Anesthesiologists physical status classification system; IMA, inferior mesenteric artery; IMV, inferior mesenteric vein; TME, total mesorectal excision

The twelve criteria mentioned formed the video assessment checklist used in this study (Fig. 1), under the following checklist subsections: (i) usability of the platform (relating to functionality of the hosting platform), (ii) video component (relating to specific audiovisual components of the videos), (iii) intraoperative techniques (the key procedural steps of the robotic TME procedure as per the EARCS consensus) and (iv) other information (content of clinical and educational significance not pertaining to the operative recording itself). Each of the twelve criteria was scored as either a 0 or 1 representing the absence or presence, respectively, of a particular criterion. Video length was also recorded for analysis.

### Video data extraction, marking and statistical analysis

Videos of robotic TME procedures were identified from the following surgical education platforms and included for analysis: WebSurg, C-SATS and Touch Surgery. These platforms were selected due to their accessibility, as they are widely available and do not require institutional access. Videos from the video sharing platform, YouTube, were also included as a counterpoint to the surgical education platforms. Videos from these platforms were reviewed independently by three reviewers (ZA, AA, HDR) and assessed using our study checklist.

Statistical analysis was performed using GraphPad Prism 10.1.1 and IBM® SPSS® Statistics 29.0.2.0. Total checklist scores, as well as individual domain scores (usability of platform, video component, intraoperative techniques and other information) and video length were compared between the surgical educational platforms and YouTube. Additionally, analyses were conducted with surgical platforms split into their respective platforms. Normality was assessed using the Shapiro–Wilk test. The Mann–Whitney *U* test was used for comparisons between surgical platforms and YouTube, while the Kruskal–Wallis test was used for comparisons among WebSurg, C-SATS and YouTube. Results are presented as median ± interquartile range (IQR). Total checklist scores, by year of upload (2016–2023), were analysed using a one-way ANOVA test. A *p* value < 0.05 was considered statistically significant for all analyses.

## Results

**Fig. 2:**
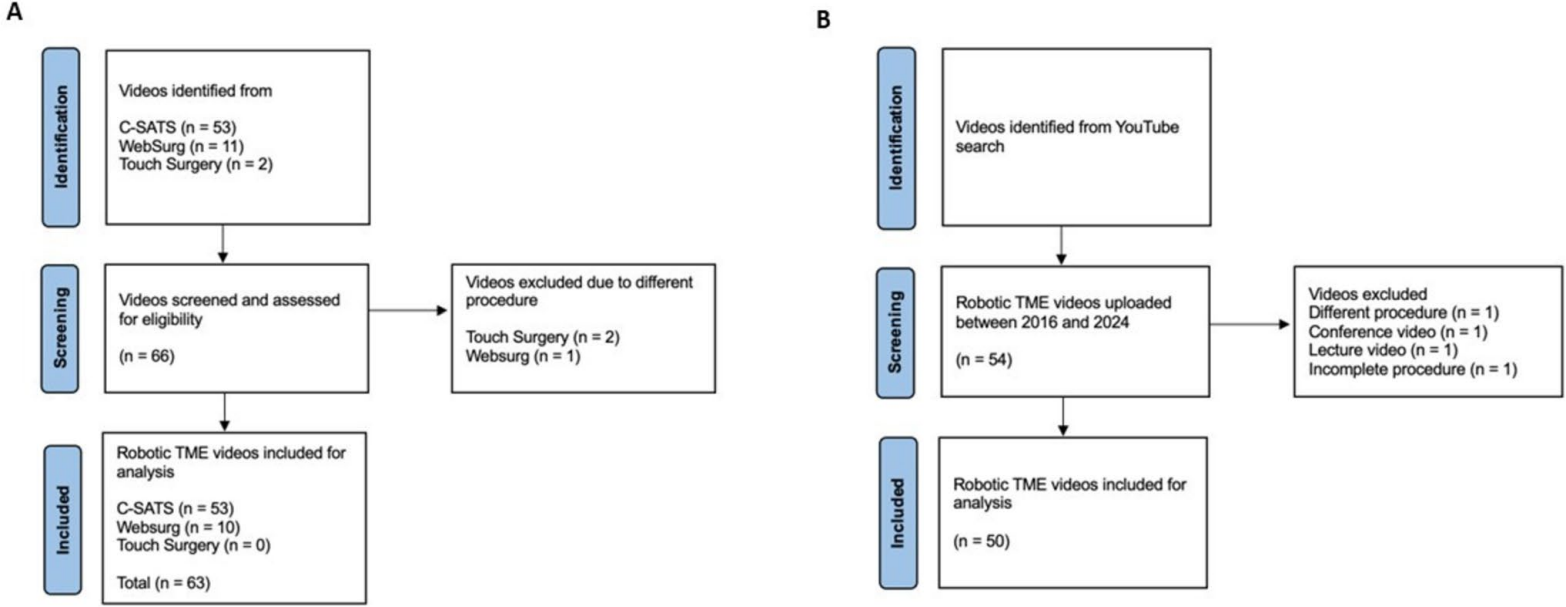
**A** Flowchart showing the selection process for videos from WebSurg, C-SATS and Touch Surgery related to robotic total mesorectal excision. **B** Flowchart showing the selection process for videos from YouTube related to robotic total mesorectal excision

A total of 120 videos were identified for screening. Flowcharts of the video search for surgical video platforms and YouTube are presented in Fig. 2 a and b, respectively. Following screening and exclusion, a total of 113 videos were analysed.

### Total checklist score

**Fig. 3:**
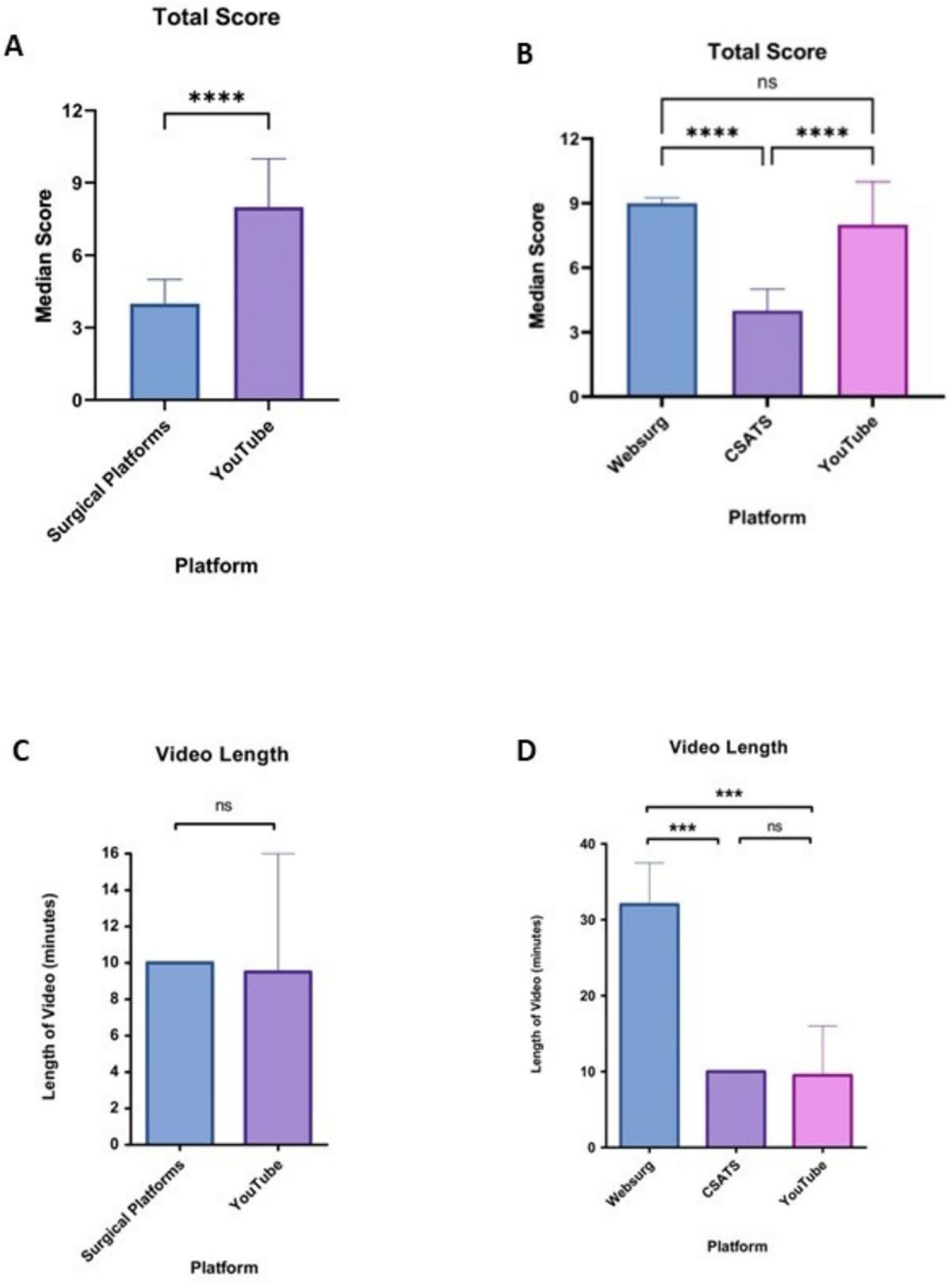
**A** Comparison of the median total score of robotic total mesorectal videos from surgical education platforms and YouTube. **B** Comparison of the median total score of robotic total mesorectal videos between WebSurg, C-SATS and YouTube. **C** Comparison of the median video length (minutes) of robotic total mesorectal videos from surgical education platforms and YouTube. **D** Comparison of the median video length (minutes) of robotic total mesorectal excision videos among WebSurg, C-SATS and YouTube

The scores and percentages of each criterion of the study checklist, displaying and comparing all extracted videos, and those from surgical platforms and YouTube are summarised in Table 1. The total median score for all surgical educational platform videos (WebSurg and C-SATS) and YouTube was 4 (4–5) and 8 (7–10), respectively. None of the videos analysed from WebSurg, C-SATS and YouTube achieved a full checklist score of 12/12. The total median checklist score obtained by YouTube videos (8 [7–10]) was significantly higher compared to surgical educational platforms (4 [4-5]) (*p* < 0.0001) (Fig. 3a). WebSurg videos had a median score of 9 (8–9), significantly higher than CSAT-S videos with a median score of 4 (4–5) (*p* < 0.0001). YouTube videos also scored significantly higher than CSATS videos (*p* < 0.0001) but were not significantly different from WebSurg videos (Fig. 3b).

**Table 1:** Study checklist and analysis of robotic total mesorectal excision videos from surgical educational platforms and YouTube

Checklist question	Total videos (*n* = 113)		Surgical educational platforms (*n* = 63)		YouTube (*n* = 50)	
	Yes (%)	No (%)	Yes (%)	No (%)	Yes (%)	No (%)
Accessibility: no login or registration is required to view the video	103 (91)	10 (9)	53 (84)	10 (16)	50 (100)	0 (0)
Easy to find video (video identifiable using key search terms)	113 (100)	0 (0)	63 (100)	0 (0)	50 (100)	0 (0)
Easy to locate specific parts of the procedure (presence of timestamps/sections/chapters)	25 (22)	88 (78)	10 (16)	53 (84)	15 (30)	35 (70)
Narration/subtitles	53 (47)	60 (53)	10 (16)	53 (84)	43 (86)	7 (14)
Educational components such as interactions, questions and visual prompts	26 (23)	87 (77)	0 (0)	63 (100)	26 (52)	24 (48)
Image quality is appropriate with constant clear view of operating field	110 (97)	3 (3)	60 (95)	3 (5)	50 (100)	0 (0)
Port site and patient position demonstrated (either diagrammatically or physically)	37 (33)	76 (67)	9 (14)	54 (86)	28 (56)	22 (44)
Robotic procedure demonstrated clearly: colonic mobilisation, clipping of IMA and IMV and TME	55 (49)	58 (51)	10 (16)	53 (84)	45 (90)	5 (10)
Anastomosis and leak test both demonstrated	19 (17)	94 (83)	4 (6)	59 (94)	15 (30)	35 (70)
Presentation of case including age, sex, ASA, indication for surgery, co-morbidities and previous imaging	58 (51)	55 (49)	24 (38)	39 (62)	34 (68)	16 (32)
Complications/30-day outcome	23 (20)	90 (80)	6 (10)	57 (90)	17 (34)	33 (66)
Video maintaining patient and surgeon confidentiality	113 (100)	0 (0)	63 (100)	0 (0)	50 (100)	0 (0)

*ASA*, American Society of Anesthesiologists physical status classification system; *IMA*, inferior mesenteric artery; *IMV*, inferior mesenteric vein; *TME*, total mesorectal excision

### Video length

The median video length across all platforms was 10 min (7–10). There was no significant difference in video length between YouTube videos (9.5 [7–16] min) and videos from surgical educational platforms (10 [6–10] min) (Fig. 3c). When further analysing the surgical platforms individually, WebSurg videos had a median length of 32 (21–35) min, which was significantly longer than C-SATS videos with a median length of 10 (6–10) min (*p* < 0.001) and longer than YouTube videos (*p* < 0.001) (Fig. 3d).

### Usability of platform

**Fig. 4:**
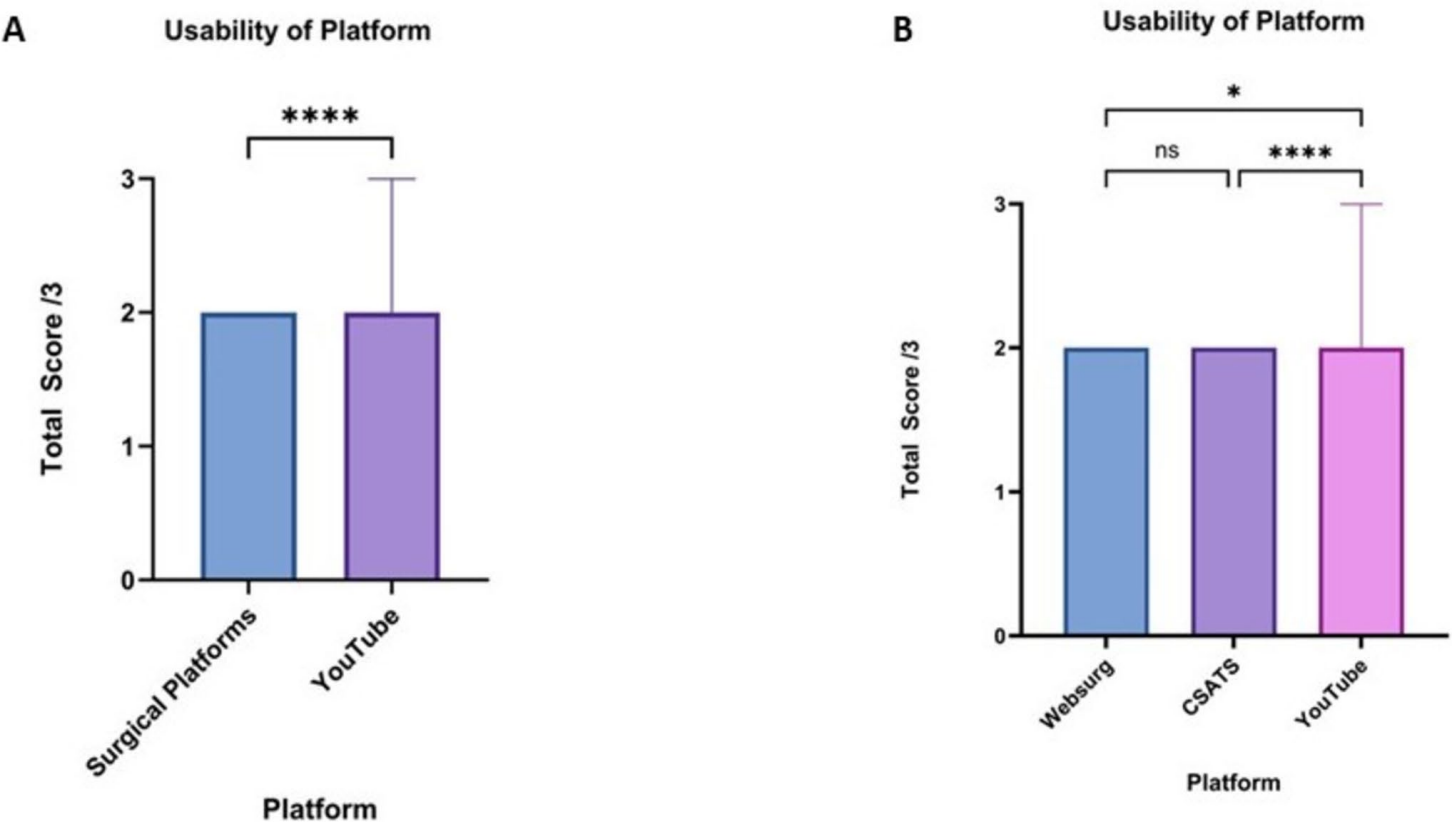
**A** Comparison of the median usability of platform domain scores of robotic total mesorectal excision videos from surgical educational platforms and YouTube. **B** Comparison of the median usability of platform domain scores of robotic total mesorectal excision videos between WebSurg, C-SATS and YouTube

The usability of platform domain scores was significantly different between surgical educational platforms and YouTube (*p* < 0.0001). The median score for surgical platforms and YouTube was 2 (2–2) and 2 (2–3), respectively (Fig. 4a). When further analysing the surgical platforms individually, WebSurg and C-SATS both had a median score of 2 (2–2). YouTube scores were significantly higher than both WebSurg (*p* < 0.05) and C-SATS (*p* < 0.0001), with no significant difference between WebSurg and C-SATS (Fig. 4b).

### Video component

**Fig. 5:**
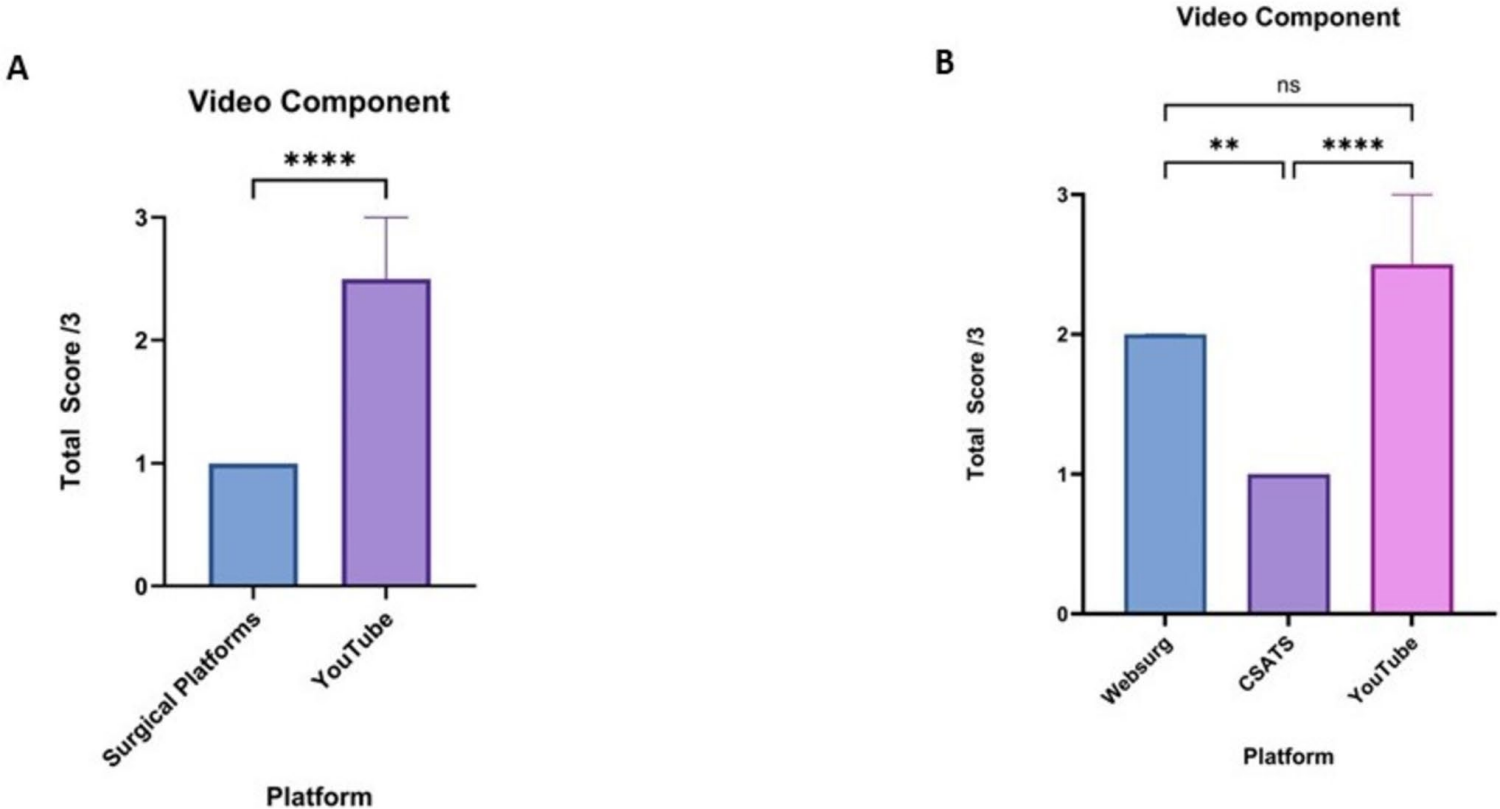
**A** Comparison of the median video component domain scores of robotic total mesorectal excision from surgical educational platforms and YouTube. **B** Comparison of the median video component domains scores of robotic total mesorectal excision between WebSurg, C-SATS and YouTube

The video component domain scores were significantly higher for YouTube compared to surgical educational platforms (*p* < 0.0001). The median score for surgical platforms was 1 (1–1), while for YouTube, it was 2.5 (2–3) (Fig. 5a). WebSurg had a median score of 2 (2–2), significantly higher than C-SATS with a median score of 1 (1–1) (*p* < 0.01). YouTube scores were higher than C-SATS (*p* < 0.0001), and there was no significant difference with WebSurg (Fig. 5b).

### Intraoperative techniques

**Fig. 6:**
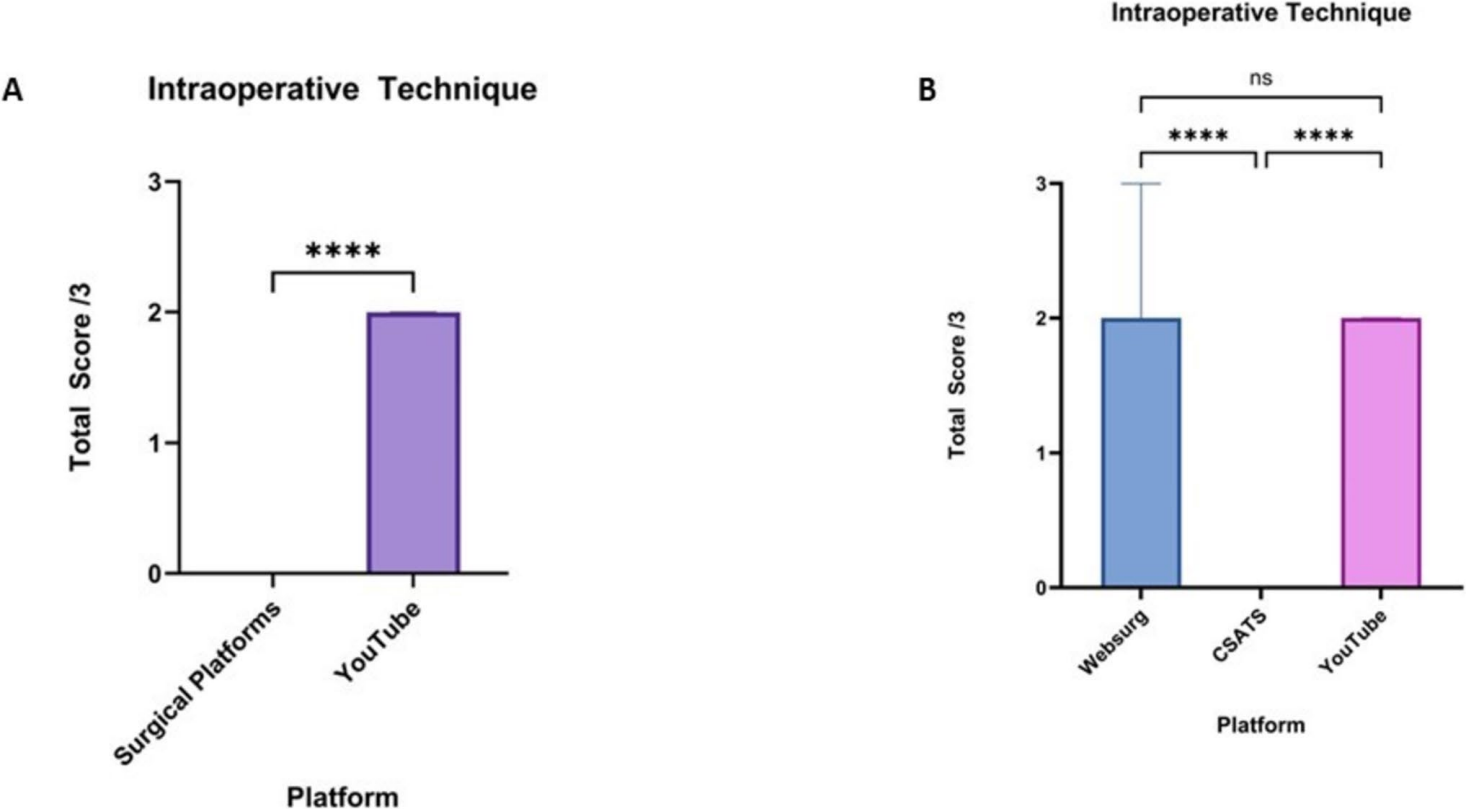
**A** Comparison of the median intraoperative technique domain scores of robotic total mesorectal excision from surgical educational platforms and YouTube. **B** Comparison of the median intraoperative technique domains scores of robotic total mesorectal excision between WebSurg, C-SATS and YouTube

The intraoperative techniques domain scores were higher for YouTube compared to surgical educational platforms (*p* < 0.0001). The median score for surgical platforms was 0 (0–0), while for YouTube, it was 2 (1–2) (Fig. 6a). When further analysing the surgical platforms individually, WebSurg had a median score of 2 (2–3), significantly higher than C-SATS with a median score of 0 (0–0) (*p* < 0.0001). YouTube scores were significantly higher than C-SATS (*p* < 0.0001) but were not significantly different from WebSurg (Fig. 6b).

### Other information

**Fig. 7:**
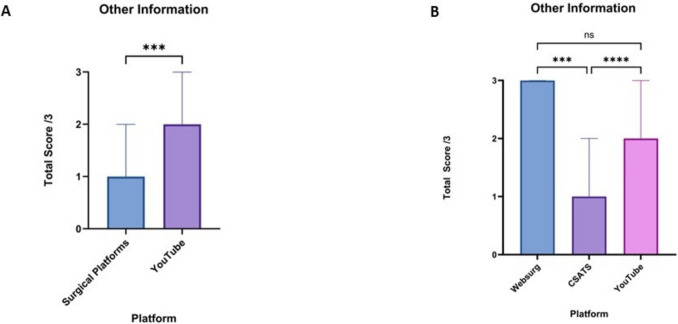
**A** Comparison of the median other information domains scores of robotic total mesorectal excision from surgical educational platforms and YouTube. **B** Comparison of the median intraoperative technique domain scores of robotic total mesorectal excision between WebSurg, C-SATS and YouTube

The other information domain scores were higher for YouTube compared to surgical educational platforms (*p* < 0.001). The median score for surgical platforms was 1 (1–2), while for YouTube, it was 2 (1–3) (Fig. 7a). When further analysing the surgical platforms individually, WebSurg had a median score of 3 (2–3), significantly higher than CSATS with a median score of 1 (1–2) (*p* < 0.001). YouTube scores were significantly higher than CSATS (*p* < 0.0001) but were not significantly different from WebSurg (Fig. 7b).

### Upload year of robotic total mesorectal excision YouTube videos

The total median checklist scores for YouTube videos were analysed by year of upload from 2016 to 2023, showing no significant differences across the years or between years (Supplementary material Figure S1). The median checklist scores per year are detailed in Table 2.

**Table 2:** Median total checklist scores according to upload year of the YouTube video

Year of upload	Number of YouTube videos	Checklist score, median (IQR)
2016	2	8
2017	5	9 (9–11)
2018	2	9
2019	6	10 (8–10)
2020	7	10 (5–10)
2021	9	8 (6–10)
2022	12	7 (6–9)
2023	6	8 (6–9)

IQR could not be calculated for the years 2016 and 2018 given insufficient data points (*n* < 4)

## Discussion

This study assessed the quality of robotic TME videos across surgical and commercial platforms using a newly designed checklist tool. We found that WebSurg and YouTube videos were not significantly different in the quality of sampled robotic TME videos both in terms of overall checklist scores and checklist component scores; robotic TME videos hosted on YouTube were higher in quality across checklist components when compared to C-SATS. In addition, users felt that the quality assessment tool created can be easily adopted for robotic TME procedures.

Multiple previous studies have compared YouTube and WebSurg using the LAP-VEGaS checklist for laparoscopic pylorotomy [21], laparoscopic myomectomy [22], laparoscopic hysterectomy [23] and thoracoscopic lobectomy [24] and found WebSurg to be superior. Other studies have made this comparison using bespoke or other checklists and found that, in the case of laparoscopic sleeve gastrectomy [25] and laparoscopic adrenalectomy [26], videos on WebSurg to be of higher educational quality than those on YouTube. Among these studies, the peer-reviewed nature of WebSurg videos is mentioned as a distinguishing aspect of the platform compared to YouTube. WebSurg videos have proven to be a high-quality, peer-reviewed resource providing expert content that meets the Health on the Net Foundation Code of Conduct requirements for quality, confidentiality and transparency [27].

One of the few studies to report little difference between surgical videos (for laparoscopic adrenalectomy) on WebSurg and YouTube, specified that ‘attentively chosen [YouTube] content … is almost as accurate as [WebSurg] content’. This study demonstrated no significant difference between YouTube and WebSurg in almost all domains of our LAP-VEGaS adapted checklist, with the platforms having overall scores of 8 and 9 out of 12, respectively. While this result may have been due to a relative improvement in YouTube videos in recent years, all above mentioned studies were published in the last 2 years, and our analysis demonstrated no significant difference in YouTube video scores per year.

When the YouTube videos included in this study were ranked in score order, it was noted that of the top 25 (50%), 56% were published by the same channel—‘Colorectal Disease Journal’, a YouTube channel associated with *Colorectal Disease* and the author(s) of the LAP-VEGaS Consensus [28]. Other high-ranking videos in this group were published by Vattikuti Foundation (a not for profit robotic surgery initiative), the *Society of American Gastrointestinal and Endoscopic Surgeons*, *Diseases of the Colon and Rectum Journal*, *AME Surgical Video DatabasE (ASVIDE)*, and several individual surgeons. This demonstrates that YouTube can be a source of high-quality educational videos provided they arise from peer-reviewed or accredited channels or individuals. However, the variability in videos on YouTube owing to the free access nature of the platform was also captured in our study, with a IQR for YouTube video total checklist scores versus those of WebSurg videos. Open-access platforms often lack peer review, leading to inconsistent educational quality [29, 30]. Critical elements such as anatomical landmarks, patient selection and complication management are frequently omitted [29]. Peer-reviewed libraries offer more standardised content, and structured accreditation of open-access videos has been proposed as a strategy to improve reliability [30, 31].

When assessing WebSurg with LAP-VEGaS or checklists adapted from it, it is important to note that the platform itself follows the LAP-VEGaS guidelines for surgical video submissions. As such, it is no surprise that videos on this platform score well across numerous studies including our own. While YouTube videos with an association to LAP-VEGaS scored well in this study, it is also worth noting that videos from channels with no stated adherence to LAP-VEGaS also produced high-scoring videos. It does not therefore appear that high checklist scores are tautologically based on stated adherence to the LAP-VEGaS criteria. While WebSurg may produce higher-scoring videos, the emergence of accredited channels producing high-quality surgical videos on YouTube makes the latter a valuable platform for surgical education, especially given its global popularity and ubiquity. The main difference between WebSurg and YouTube videos was length. In our study, the median duration of YouTube videos was 9.5 min, compared to 32 min for WebSurg videos. Prior research suggests that surgical trainees prefer videos of approximately 10 min in length [32], with engagement tending to decline significantly for longer videos [33]. These findings highlight the need to optimise surgical video length to maintain trainee engagement. While shorter, high-yield videos may align with newer generations [34], longer, more detailed videos may still be valuable by providing comprehensive demonstrations of operative techniques and supporting deeper understanding of procedural steps [11].

This is the first study, to our knowledge, to assess and compare C-SATS surgical videos in regard to their educational value. Based on this specific checklist, we found that checklist scores for C-SATS were lower than YouTube across all checklist domains except platform usability. C-SATS is a platform in which surgeons upload videos of their own operations which are then peer-reviewed in text comments and rated and has been previously utilised in studies assessing the quality of surgical residents based on videos of operative tasks they were set [35]. As such, videos on these platforms are brief, technical, anonymised and blinded—with no narration or context. Inclusion of this platform in our study was useful, as it demonstrated the efficacy of our checklist with nearly all videos scoring lower than WebSurg and YouTube.

All three platforms showed no significant difference in their platform usability scores, suggesting that most video hosting platforms are technologically up to date and include robust search functionality and video segmentation, with the differences between the platforms mostly arising from the quality of the video content itself. Despite being developed primarily for laparoscopic surgery, our adapted quality assessment checklist is easy to apply to robotic surgery videos. While studies using the original LAP-VEGaS checklist appraise videos based on the ‘demonstration of the surgical procedure’ criteria, we opted to specify key elements of the robotic TME as set forth by the EARCS consensus [20]. A similar approach was adopted by our collaborators Alghazawi et al. [16] for the evaluation of laparoscopic sleeve gastrectomy (LSG) videos by developing their checklist in accordance with the Bariatric Metabolic Surgery Standardisation consensus for LSG [36]. This approach provides a further layer of validation to the process of appraising surgical educational videos and ensures that high-quality materials are made available to surgeons and surgical trainees. Such validation of videos of clinical intervention is much needed—a recent scoping review showed that there is significant heterogeneity and poor quality control in clinical intervention videos in peer-reviewed literature [37]. The SPRINT (Standards for Presenting and Reporting clinical InterveNtions Televisually) reporting guidelines are currently under development as a universal checklist for all clinical interventional videos, registered within the EQUATOR (Enhancing the QUAlity and Transparency Of health Research) Network [38]. The aim of SPRINT is to improve the reliability and quality of published video research by promoting transparency and quality of surgical videos in the literature.

While this study has focused on the evaluation of traditional video resources, it is important to acknowledge the multitude of emerging technologies reshaping surgical education—virtual reality (VR)-based simulation allowing for high fidelity, immersive and realistic operative training [39, 40]; point-of-view video capture using wearables such as head-mounted cameras and more recently smart glasses [41, 42]; and artificial intelligence (AI)-driven video/VR resource creation, annotation/overlay and trainee feedback [43]. However promising these tools are, it is important to continue building validated assessment frameworks and to stay vigilant of their potential inaccuracies [44]. Despite checklist standardisation, subjectivity in reviewer scoring remains a limitation. Structured reviewer training and the use of standardised tools in national laparoscopic programmes have improved assessment consistency [45]. Objective methods such as AI-driven phase detection may further enhance scoring reproducibility [43].

### Strengths and limitations

The main strength of this study includes adaptation of a validated appraisal tool [14], and three group comparisons with a particularly high number of videos from C-SATS and YouTube. A significant limitation of this study is the relatively low number of WebSurg videos appraised, along with no videos from Touch Surgery identified. In addition, we identified videos that were across surgical and commercial platforms that did not require institutional access. Despite the overlap, we did not specifically appraise peer-reviewed literature or videos that went through a strict peer review process prior to upload.

## Conclusions

This study demonstrated variability in the educational quality of online robotic TME videos based on our quality assessment checklist. Platforms such as a WebSurg and YouTube were comparable, with videos from YouTube being significantly longer on average than WebSurg. Videos from C-SATS scored significantly lower when compared to WebSurg and YouTube based on the domains of our quality assessment checklist. Overall, the usability of all three platforms was equivalent, and the main differences between them arose from the content and presentation of the videos themselves. These findings suggest that there is a need for guidelines which will provide a set of minimum items that should be reported by clinicians when uploading surgical videos. This will ultimately ensure high-quality and technically accurate videos and lead to a more complete and transparent reporting of videos by health researchers, thus facilitating evidence-based decision-making and reproducible research.

## Supplementary Information

Below is the link to the electronic supplementary material.

Supplementary file1 (DOCX 96 KB)

## Data Availability

No datasets were generated or analysed during the current study.
