# Supplementary material for: Development of an evaluation framework for robotic total mesorectal excision videos: a review and comparison of medical professional and public video resources
Source: Int J Colorectal Dis. 2025 May 24;40(1):127. doi: 10.1007/s00384-025-04914-w (PMC12103343; doi:10.1007/s00384-025-04914-w)
Supplement: Supplementary file 1 — Supplementary file1 (DOCX 96 KB) [file 384_2025_4914_MOESM1_ESM.docx]

**Supplementary material**


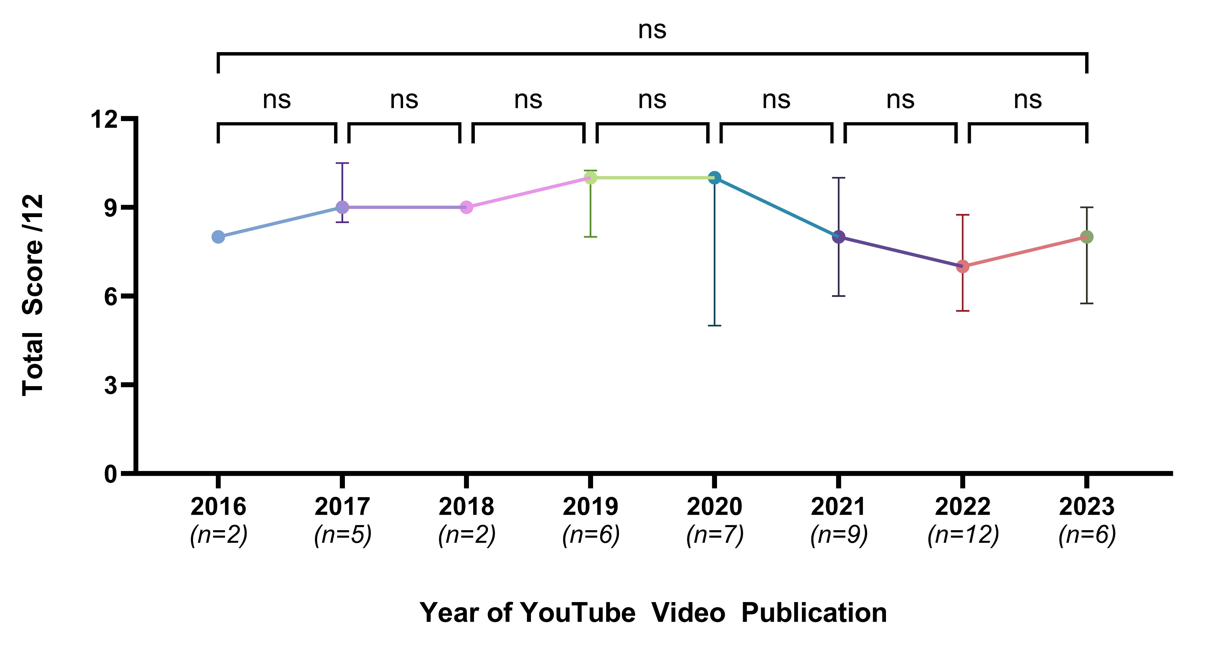


**Fig. S1.** The total median checklist score of YouTube videos per year of video upload.
